# Society Proceedings

**Published:** 1898-11

**Authors:** 


### SOCIETY PROCEEDINGS.

### A. V. M. A.

THE county organization of Greater New York has sent in a unanimous call for the Association to meet there in 1899. The State organization adjourned in September to meet in Ithaca in 1899, unless the national organization convenes in New York City, under which circumstances the State meeting will convene in the Metropolitan City. Editors Bell, of the *Review*, and Gill, of the JOURNAL, strongly urge the attractions of "Gotham."

The District of Columbia Veterinary Association has passed resolutions urging the Association to convene in the Capitol City, and other organizations of the city will take similar action in the interest of the movement.

Detroit, through her Convention City organization, sends a strong list of attractions why our organization should convene there in 1899. Are her veterinarians speaking through the city's official representatives?

### MAINE VETERINARY MEDICAL ASSOCIATION.

THE regular meeting was held at the Elmwood House, Waterville, October 13th, at 7.30 P.M., President West in the chair. Present, Drs. West, Stevens, Jolly, Purcell, Freeman, Russell, and Salley. The minutes of the previous meeting were read and approved.

It was decided to make a strong effort to pass a veterinary registration bill this winter. Previous efforts at the passage of such a bill have failed for various reasons, the chief of which are a lack of coöperation of members of the Association and a misunderstanding of the object and real meaning of the bill. On motion of Dr. Stevens, seconded by Dr. Purcell, it was voted to instruct the Secretary to send a circular explanatory letter to the non-graduates practising in the State, together with a copy of the proposed bill. It is believed that there will be little opposition to this bill if it is well understood.

Dr. Stevens read a paper upon "Puerperal Septicæmia," which was very interesting, and considerable discussion followed.

Voted to adjourn to meet at Augusta in January.

I. L. SALLEY, D.V.S.,
Secretary.

### VETERINARY MEDICAL SOCIETY, UNIVERSITY OF PENNSYLVANIA.

THE first meeting of the year was held October 14th, at 8 o'clock. The Secretary, Mr. Jacobs, called the meeting to order, and Mr. Nolan was appointed Secretary *pro tem.* Mr. Hoopes was made critic. The names of Messrs. Kerns, Tallman, Mayer, Young, Bader, Walter, Watson, and Gilliland were proposed and duly elected to membership.

The following gentlemen were elected as officers: Mr. Jacobs, President; Mr. Taylor, Vice-President; Mr. Hoopes, Treasurer; Mr. Nolan, Secretary, and Mr. Hughes, Librarian. Executive Committee, Messrs. Kerns, Comman, Tallman, and Bader.

Meeting adjourned at 9.15 P.M.

Second meeting was held October 21st, at 8 o'clock. The Society was called to order by President Jacobs. Mr. Young was appointed critic. The names of Messrs. Powell, Dunn, and Shore were proposed and duly elected to membership.

Prof. Leonard Pearson then gave an interesting, likewise instructive, address on "The Conformation of the Horse."

A vote of thanks was extended to Prof. Pearson for his valuable address.

Meeting adjourned at 9.30 P.M.

Third meeting was held November 4th, at 8 o'clock. The Society was called to order by the President. Mr. Kerns was appointed critic. The names of Messrs. Colton, Woodward, Bender, Notton, and Willett were proposed and elected to membership.

Literary programme was as follows: A paper by Mr. Hoopes on "Male Animals in the Stud."

DEBATE. *Resolved*, That a veterinarian should not have his office at a livery-stable. *Affirmative:* Messrs. Cheney, Young, Walter. *Negative:* Messrs. Keiter, Hughes, Gilliland. The subject was ably handled by the gentlemen in the debate; likewise by the members in general. The negative side was victorious.

Meeting adjourned at 9.30 P.M.

L. A. NOLAN,
Secretary.

### VETERINARY MEDICAL ASSOCIATION OF NEW YORK COUNTY.

THE regular monthly meeting of the Association was called to order at 8.45 P.M. on Wednesday, November 2, 1898, with the President, Dr. Robertson, in the chair. The following members responded to roll-call: Drs. Amling, Ackerman, Bell, J. S. Cattanach, J. S. Cattanach, Jr., Dickson, Ellis, Foy, Gill, Grenside, Hanson, MacKellar, O'Shea, Robertson, and Ryder (15). The minutes of previous meeting were read and approved.

Dr. Gill, Chairman of the Board of Censors, offered the following report: Charges have been preferred against Dr. J. S. Lamkin for violation of Article VII. of the Code of Ethics, he having connected himself with a livestock insurance company. On motion of Dr. Bell, seconded by Dr. Cattanach, the chairman was requested to notify Dr. Lamkin to present himself to the Board of Censors at their next monthly meeting, to answer said charges; carried. Signed, Dr. Gill, chairman; Dr. Bell, Dr. Cattanach, Dr. MacKellar. Moved and seconded that the report of the Board of Censors be accepted and placed on file; carried.

Dr. O'Shea, chairman of the Judiciary Committee, stated that although it was a little early in the season for legislative work, the committee had been advised that several bills at variance with our present State veterinary laws were on foot, but that the Judiciary Committee were on the alert to attack them when introduced.

Dr. Grenside read a very interesting and practical paper entitled "The Horse's Mouth," which was freely discussed, Drs. Bell, J. S. Cattanach, and Ackerman leading in the discussion. Motion for a vote of thanks to be tendered to Dr. Grenside for his paper was made by Dr. O'Shea; seconded; carried.

Dr. E. B. Ackerman read a paper entitled "Municipal *versus* State Control of Tuberculosis." Dr. Ackerman's paper opened a field for discussion, which was led by Dr. Gill and participated in by Drs. Bell, Hanson and others. Moved and seconded that a vote of thanks be extended to Dr. Ackerman; carried.

Dr. Bell, chairman of Ways and Means Committee, reported that he had furnished two excellent papers for the present meeting, as was evidenced by their reading, and had one by Dr. Ryder promised for the December meeting, and would secure a second one from among the members. Moved and seconded, that the report be accepted; carried.

Moved and seconded, that a committee be appointed to draft resolutions on the death of our late member, Dr. Machan; amendment, that Dr. Robertson be included in that committee; carried. The President appointed the following committee to act in that capacity, Dr. H. D Hanson, chairman, Dr. James L. Robertson, Dr. J. Elmer Ryder.

Under the head of new business, Dr. Gill raised the question of the American Veterinary Medical Association holding its next meeting in New York City. After some discussion it was regularly moved and seconded that the Secretary be instructed to notify the chairman of the executive committee of the national body that it was the sense of this meeting that the A. V. M. A. be invited to hold their next annual meeting in New York City. Moved, by Dr. Gill, that the vote be made unanimous; seconded; carried.

Moved by Dr. Hanson, that the Secretary, in conjunction with the Ways and Means Committee, get up a circular letter, to enthuse a full membership at the annual meeting, the yearly announcement of each member's indebtedness to the Association to accompany the letters; seconded; carried.

Moved and seconded, that the meeting adjourn; carried.

ROBERT W. ELLIS, D.V.S.,
Secretary.

### OHIO STATE BOARD OF VETERINARY EXAMINERS.

A MEETING was called at the State House, Columbus, October 6th, 1898. All members present. After the usual routine reading and approval of the minutes of the last meeting the following motions prevailed and were passed by the Board. Moved by Dr. Probst, seconded by Dr. Wight, that certificates issued by the Pennsylvania State Board of Veterinary Medical Examiners be accepted upon the same terms as certificates of the Ohio Board are accepted by the Pennsylvania Board, and that notice be given by the respective boards of the acceptance of such certificates.

On motion by Dr. White, the certificate presented by one Dr. A. E. Cunningham, from the Pennsylvania Board, be accepted in lieu of an examination, and that a certificate be issued him by the Ohio Board. Beside the mere presentation of a certificate from the State of Pennsylvania, a letter from Dr. Hoskins, of the Pennsylvania Board, highly recommending Dr. Cunningham, was read by Dr. White.

As this was a meeting to consider prosecutions, it was moved by Drs.

Probst and White that when complaints are hereafter received by the Board that some person is illegally practising veterinary medicine and surgery in the State of Ohio, the Secretary of the Board demand of the informant proof of the charges, and, upon receipt of such proof, shall furnish the prosecuting attorney of the county in which such illegal practitioner resides with a copy of the charges, and request prosecution.

Just before adjournment, the newly appointed member of the Board, Dr. D. S. White, of the Ohio State University, Veterinary Department, was elected Secretary.

DAVID S. WHITE,
Secretary.

### MISSOURI VALLEY VETERINARY ASSOCIATION.

THE seventeenth regular meeting of the Association convened in the lecture-hall of the Kansas City Veterinary College on the evening of October 5, 1898. President Bennett presided, and on roll-call the following members responded: Drs. S. E. Bennett, S. Stewart, J. H. Cock, B. F. Kaupp, J. C. Milnes, R. P. Steddom, S. L. Hunter, R. C. Moore, W. A. Heck, and Chas. Saunders. Visiting members of the profession were H. H. George, J. S. Buckley, and about twenty veterinary students. The customary disposition was made of the minutes of the previous meeting.

The Anti-vivisection Committee reported, and on motion the report was accepted and the committee discharged.

Dr. L. M. Klutts' request for associate membership was refused upon the grounds that the doctor had resigned a few years since when he was in arrears for dues; also that the letter-head upon which the request was written contained the cut of a horse, which is a violation of the Code of Ethics of this Association.

Within a few months several of our members have moved out of our territory. Dr. G. A. Johnson, transferred from Kansas City to Sioux City, Ia.; R. H. Harrison to Milwaukee, Wis., and Nelson S. Mayo to Storrs, Conn. Each of these gentlemen presented his resignation to the Association, and, being reported with clean accounts, on motion their resignations were accepted and they were placed upon our honorary list.

The Secretary reported collections very much improved, but there were several members still who were very slow in settling accounts. The usual amount of discussion of this subject was done, and, finally, instruction was given to the Secretary to notify each individual in arrears two years or more that unless his account be settled by the next regular meeting final action will be taken.

Dr. S. L. Hunter read a paper on "Army Practice," which was listened to with much interest. He added another chapter to the very thorough but much-needed airing of the condition of the army veterinarian.

Dr. J. C. Milnes responded to the theme, "The Reason Why," and described several outbreaks of anthrax in horses, relating the origin of such, and expressed his wonder why there are not more of such outbreaks, considering the unsanitary condition of many barns and stables. Then followed some spirited but good-natured criticisms of the author's theories of etiology and diagnosis. In defence, the author went carefully over the train of symptoms and postmortem lesions, and stated he had been in practice since 1878, and had seen much anthrax, and felt reasonably sure of his diagnosis. Those who took the negative side of the question went into the bacteriological aspect of the disease and stated that even a microscopical examination will not always substantiate a suspicion. The listeners were impressed with the idea that we should guard our opinions in such cases until we are absolutely sure of our diagnosis, as there are so many factors to be considered that error is easily mistaken for truth.

Dr. B. F. Kaupp read a carefully prepared paper on "Calculi." He entered into detail of the formation and composition of calculi of all descriptions. The exhibition of a fine collection of calculi added much interest to the subject. Dr. Cock created considerable merriment by relating the story of the Irishman insisting that calculi are contagious. Dr. Hunter cited a case where a leather shoestring was extracted from the bladder of an old man; the patient was unable to explain how the queer structure came there.

Dr. Stewart's paper on "The Advantages of Active Membership in a Veterinary Association" was listened to in silent admiration. Not only was the subject-matter very practical and inspiring, but the euphonious expressions and rhetorical phrases were so pleasing that all present were sorry when he had finished. It is to be hoped that those who were fortunate enough to hear it were sufficiently enthused to resolve to do more association work in the future.

Then came case reports.

Dr. Moore reported an injury to a bull's penis, which became so bad that the animal was destroyed. The injury at first did not seem so bad, but gradually the adjacent parts became involved—swelling and sloughing till a very large cavity was left. No cause could be given, and snake-bite was suggested. The symptoms of snake-bite were thoroughly discussed. Dr. Moore also reported a case of what he thought to be "gangrenous dermatitis" in a horse. He had seen several cases of late years in his practice, all of which had proved fatal. This does not seem to be a common disease in this section, and but few of the members had seen anything like it.

One of the interesting features of the meeting was the exhibition of a new thermo-cautery, an invention of one of our own members. This invention has the advantage of being so simple that it can be pumped with the same hand which holds the instrument while at work, also the cost of the whole instrument is not more than five dollars.

The next meeting was voted to be held in St. Joseph, Mo.

W. A. HECK.
Secretary.

